# COMPASSIONATE LOVE AND LONELINESS: LATER LIFE MENTAL HEALTH IN THE UNITED STATES

**DOI:** 10.1093/geroni/igac059.1896

**Published:** 2022-12-20

**Authors:** Nirmala Lekhak, Tirth Bhatta, Eva Kahana, Joel Synder

**Affiliations:** University of Nevada, Las Vegas, Nevada, United States; University of Nevada Las Vegas, Las Vegas, Nevada, United States; Case Western Reserve University, Cleveland, Ohio, United States; University of Nevada, Las Vegas, Nevada, United States

## Abstract

Loneliness is a serious public health problem that affects over 25% of older adults and is associated with an increased risk of depression, cognitive decline, and premature death. Previous research on social support mechanisms that contribute to loneliness has consistently illustrated the role of emotional support in reducing loneliness. However, the importance of compassionate love in reducing loneliness and, as a consequence, improving psychological well-being in later life has received little attention. Neurobiology indicates that the brain regions associated with loneliness and compassion overlap, suggesting that increasing compassion-related emotions may help alleviate loneliness. Using data from a nationwide web-based survey (n=1,861), we examined the influence of compassionate love on loneliness and assessed whether loneliness mediates the relationship between compassionate love and mental health outcomes. Even after controlling for emotional support, estimates from an ordinary least squares regression (OLS) model suggest that older adults who felt loved had significantly lower levels of loneliness (b=-0.84, p< 0.001). Feeling of love also contributed to significantly fewer depressive symptoms (b=-2.03, p< 0.001) and anxiety (b=-1.07, p< 0.001). Loneliness completely mediated the effect of compassionate love on anxiety (b=-0.82, p< 0.001) and significantly mediated its influence on depressive symptoms (b=-1.18, p< 0.001). Our findings underscore the need to design interventions that increase compassionate love to reduce loneliness and improve psychological wellbeing among older adults.

